# Autoimmunity in Acute Myocarditis: How Immunopathogenesis Steers New Directions for Diagnosis and Treatment

**DOI:** 10.1007/s11886-020-01278-1

**Published:** 2020-03-20

**Authors:** Karina Bruestle, Klaus Hackner, Gudrun Kreye, Bettina Heidecker

**Affiliations:** 1 Columbia Center for Translational Immunology, Columbia University College of Physicians and Surgeons, New York, NY USA; 2 Department of Pneumology, University Hospital Krems, Karl Landsteiner University of Health Sciences, Krems, Austria; 3 Department of Internal Medicine II, Vienna General Hospital, Division of Cardiology, Medical University of Vienna, Vienna, Austria; 4 Division of Palliative Care, Department of Internal Medicine 2, Karl Landsteiner University of Health Sciences, University Hospital Krems, Krems, Austria; 5 Division of Cardiology, University Hospital Berlin, Charite, Campus Benjamin Franklin, Berlin, Germany

**Keywords:** Myocarditis, Autoimmunity, Immune checkpoint inhibitors

## Abstract

**Purpose of Review:**

Over the last decade, myocarditis has been increasingly recognized as common cause of sudden cardiac death in young adults and heart failure overall. The purpose of this review is to discuss hypothesis of development of non-infectious myocarditis, to provide a description of the immunopathogenesis and the most common mechanisms of autoimmunity in myocarditis, and to provide an update on therapeutic options.

**Recent Findings:**

A new entity of myocarditis is immune checkpoint inhibitor (ICI) induced myocarditis. ICIs are used in advanced cancer to “disinhibit” the immune system and make it more aggressive in fighting cancer. This novel drug class has doubled life expectancy in metastatic melanoma and significantly increased progression free survival in advanced non-small-cell lung cancer, but comes with a risk of autoimmune diseases such as myocarditis resulting from an overly aggressive immune system.

**Summary:**

Myocarditis is an inflammatory disease of the heart with major public health impact. Thorough understanding of its immunopathogenesis is crucial for accurate diagnosis and effective treatment.

## Introduction

Myocarditis refers to an inflammatory process in the heart that can be initiated by various factors. The most common cause of myocarditis is viral infection [1]. However, other factors such as systemic autoimmune disease, toxins, or hypersensitivity to medications may induce myocarditis through an autoimmune reaction by various mechanisms. Even in viral myocarditis, an autoimmune reaction such as antigen mimicry may be induced. A novel cause of myocarditis is immune checkpoint inhibitor (ICI)-induced myocarditis, a rare but severe complication in this evolving field of therapy in oncology.

In this review, we will describe the pathophysiology of autoimmunity in myocarditis. A specific focus will be on ICI-induced myocarditis. This review will not discuss diagnostic approaches or prognostic features but focus on pathogenesis of autoimmune processes and link them to therapeutic strategies. A thorough understanding of the pathophysiology of ICI-induced myocarditis and other subtypes of myocarditis will be necessary to develop effective therapies.

### Definition, Etiology, and Epidemiology

Acute myocarditis is defined as an acute inflammatory disease of the myocardium, caused by a variety of infectious (e.g., viral, bacterial) and noninfectious conditions (including cardiotoxins, hypersensitivity reactions, systemic disorders, and radiation). The list of possible causal agents is constantly expanding and recently immune checkpoint inhibitors (ICI), a new class of paradigm-shifting immune-oncologic therapies was found to have potential cardiotoxic properties by triggering myocarditis [2]. The ESC working group on myocardial and pericardial diseases recommends distinguishing between viral myocarditis, autoimmune myocarditis, and viral and immune myocarditis [3]. Acute myocarditis is defined as a new-onset of symptoms (days up to 3 months) or worsening of symptoms, whereas subacute and chronic myocarditis is defined as having symptoms for more than 3 months [3].

Due to the absence of a sensitive noninvasive diagnostic test, no comprehensive population–based epidemiological data exist about the prevalence, or presenting symptoms of various etiologies as of today. However, early studies suggest that cardiac involvement may occur in 3.5 to 5% of patients during outbreaks of coxsackievirus [4]. Also, cardiac magnetic resonance imaging studies (CMR) have shown that myocarditis continues to be underdiagnosed and that broader CMR screening may be necessary to identify patients with less aggressive forms of myocarditis [5]. PVB19 is the most frequent virus detected by PCR analysis. However, similar percentages of PVB19-positive analysis have been demonstrated in patients with non-inflammatory cardiomyopathy undergoing cardiac surgery questioning the role of PVB19 persistence as pathogenic agent and suggesting it may be an innocent bystander [6]. Due to PCR amplification of viral genomes, other viruses (such as adenovirus, Epstein-Barr, and influenza virus) have been identified, but the pathophysiological and prognostic significance is still uncertain [7, 8]. Other infectious causes of myocarditis include *Trypanosoma cruzi*—a protozoan parasite causing Chagas disease, and bacteria such as group A streptococcus. An often overlooked cause for myocarditis is hypersensitivity to medications (such as dobutamine or phenytoin [9]) or drugs (such as methamphetamine or cocaine [10]). Myocarditis may also be found on endomyocardial biopsies (EMBs) amongst patients with stress-induced or Takotsubo cardiomyopathy [11]. The most aggressive forms of non-infectious myocarditis are giant cell myocarditis and eosinophilic necrotizing myocarditis, which are frequently lethal despite maximal medical treatment. A new entity is ICI-induced myocarditis, which is a result of an “unleashed” immune system with high mortality [2].

In general, many cases of myocarditis are likely underdiagnosed due to subclinical or nonspecific symptoms [5, 12]. On the other hand, subtle cardiac symptoms may be overshadowed by systemic manifestations of severe underlying infections. An analysis of national inpatient sample data from 2005 to 2014 in the USA concluded a gradual increase of reported cases of myocarditis from 95 per 1 million in 2005 to 144 per 1 million in 2014 [13]. Overall in-hospital mortality was reported to be 4.43% with a significant increase of cardiogenic shock from 6.95% in 2005 to 11.99% in 2014 [13]. Another study included data from the USA on 27,129 hospitalizations with discharge diagnosis myocarditis from 2007 to 2014. Cardiogenic shock and ventricular fibrillation/cardiac arrest occurred in 6.5% and 2.5%, respectively, with females being more affected than males [14]. The global incidence of myocarditis in 2017 as reported by the Global Burden of Disease project was 3,071,000 cases [15]. The incidence of ICI myocarditis may be up to 1–2% with significant mortality (30%) [16^•^]. Combination of ICI treatment increases the risk of myocarditis as compared with single drug therapy. Given the early success of ICI in advanced cancer [17], we expect to see an increase of autoimmune diseases related to this novel drug class in the future [18].

## Most Common Mechanisms of Autoimmunity in Myocarditis

### Pathophysiology of an Exaggerated Immune Response

**Fig. 1:**
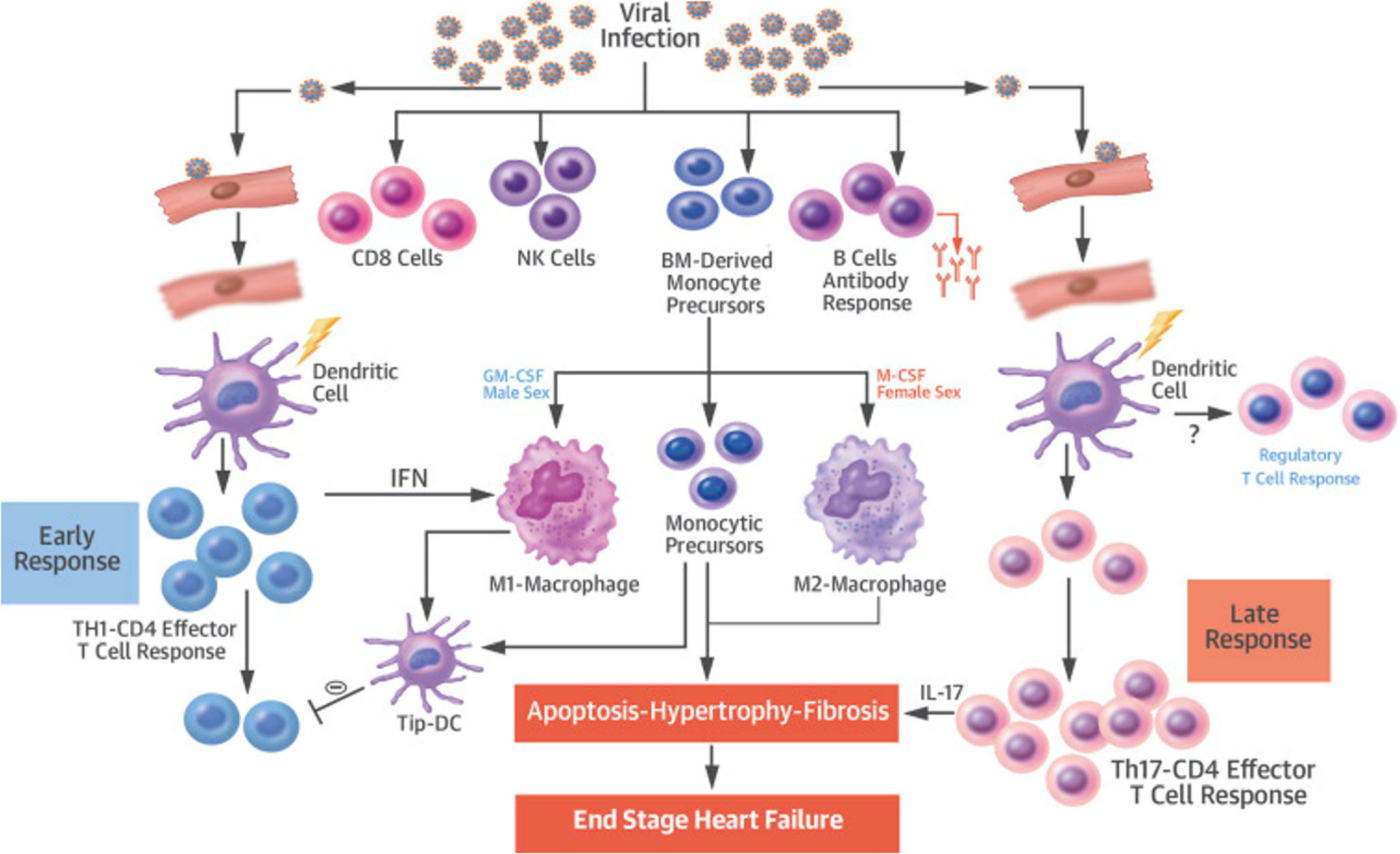
Inflammation is driven by Th-1 cells in the early phase, with M1 macrophages playing an important role. Late response is characterized by M2 macrophages and Th17 response. IL-17 is a key cytokine in progression to dilated cardiomyopathy. BM = bone marrow, DC = dendritic cells, GM-CSF = granulocyte-macrophage colony stimulating factor, IFN = interferon, IL = interleukin, M-CSF = macrophage colony-stimulating factor, NK = natural killer, Tip-DC = tip-dendritic cell (reproduced from Heymans et al. [19•])

Since the most studied inciting factor of myocarditis is thought to be viral infection, this section will focus on the pathophysiology of myocarditis after an acute viral infection, which has been conceptualized as a multiphase model recently reviewed by Heymans and colleagues (Fig. 1) [19^•^]. Initially, acute injury may be caused by direct cytotoxicity to the myocardium through viruses and other pathogens, while inflammatory molecules such as cytokines released during the immune response lead to a cascade of cytolysis, additional recruitment of inflammatory cells, and remodeling [20]. It is believed that multiple cellular and extracellular compartments of the myocardium and the classical innate and adaptive immune system contribute to effector and regulatory influences that shape clinical presentation.

### Macrophages: Microbicidal and Regulatory Cells in Cardiac Inflammation

**Fig. 2:**
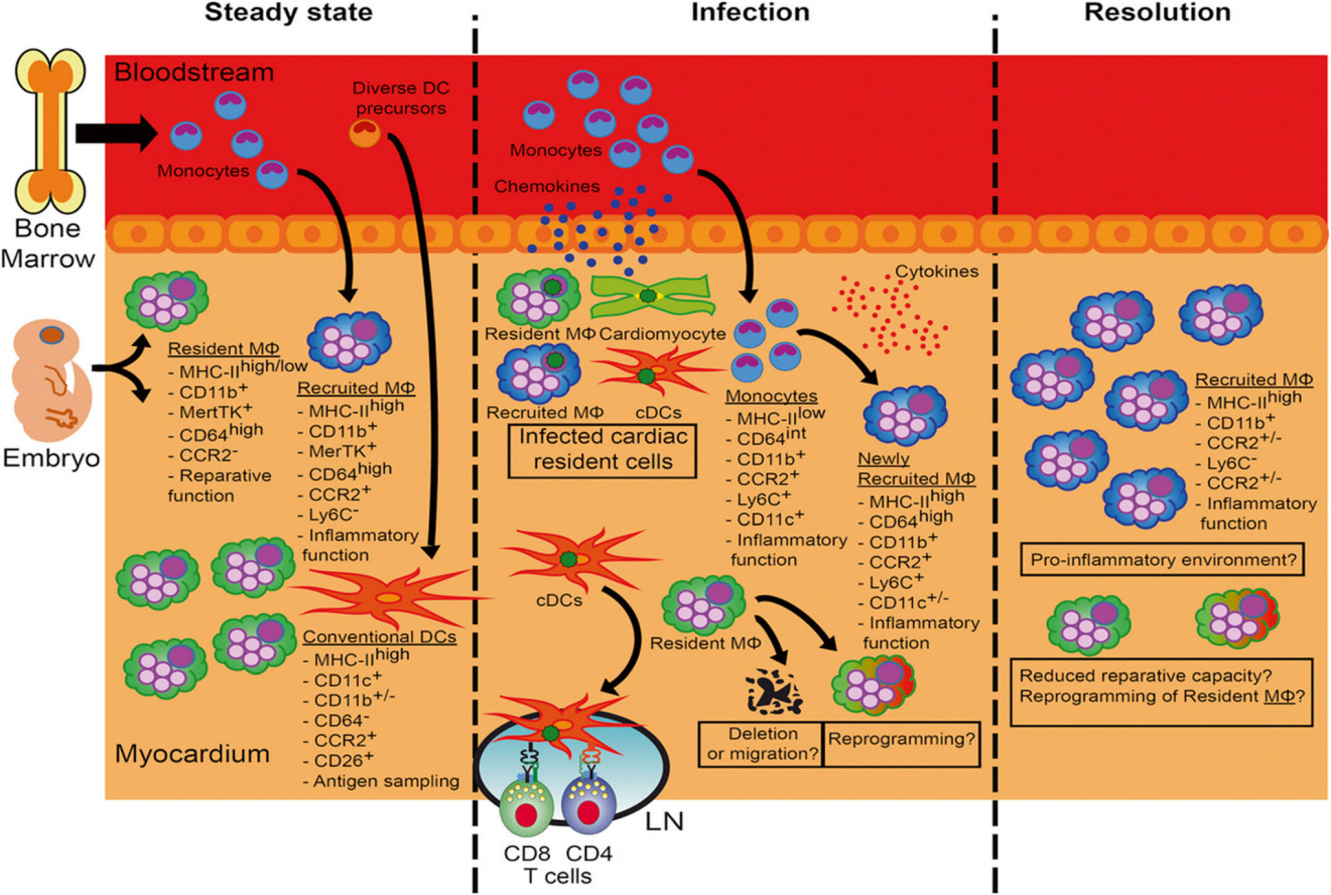
The role of macrophages in viral myocarditis: In healthy myocardium, 2 ontologically different types of macrophages can be identified in the heart (M Φ). During viral infection, cells residing in the myocardium produce chemokines to attract monocytes, which subsequently turn into macrophages with proinflammatory function. The NLRP3 pathway genes expressed by recruited monocytes have been shown to play a central role in this inflammatory process leading to delivery of interleukin-1 beta [33]. Complete depletion of macrophages in viral myocarditis is associated with increased mortality. In contrast depletion of macrophages in the chronic phase of EAM was associated with less fibrosis, which was possibly beneficial for outcomes (reproduced from Lavine et al. [21])

The majority of immune cells found in human and experimental myocarditis are of monocyte or macrophage lineages (Figs. 1 and 2) [19^•^, 21]. Cardiac injury as a result of myocarditis results in early recruitment of Ly6C^hi^ inflammatory macrophages [22, 23]. In line with these findings, blockade of chemokines associated with the recruitment of Ly6C^hi^ positive monocytes, such as CCR2 ligands CCL2/MCP1 or CCL3/MIP1α, improves autoimmune processes in myocarditis [24, 25].

Differentiation of monocytes into M1 macrophages, which have proinflammatory characteristics, is strongly influenced through CT4^+^ T cells [26]. IFN-γ produced by Th1 cells potentiates microbicidal activity of macrophages and antigen presentation [27]. This mechanism of strengthening the immune response is likely beneficial to a certain degree but can be harmful and cause “collateral damage” if it leads to an overwhelming immune reaction—a phenomenon often observed in autoimmune diseases.

M2 macrophages on the other hand decrease inflammatory response and promote fibrosis and healing in the myocardium through Ly6C^low^ activation [28, 29]. In addition, they can be activated through IL-4 and -13 secreting Th2-cells [30]. During the transition of acute myocarditis to chronic pathological remodeling, these macrophages are replaced by myofibroblast with profibrotic features [31, 32]. As macrophages play a critical role in cardiac healing with respect to cardiomyocyte death and remodeling, there has been a growing interest in modifying them for therapeutic purposes in cardiovascular disease, in particular, in myocarditis and after myocardial infarction [33].

## Hypothesis of the Development of Noninfectious Myocarditis

### Specific Trigger Factors

Myocarditis of noninfectious origin may develop as isolated cardiac disease or may be associated with a wide spectrum of systemic autoimmune diseases [34]. In contrast to viral myocarditis, the exact trigger for autoimmune myocarditis is unknown. External trigger factors include drugs such as antibiotics (ampicillin, azithromycin, cephalosporins, tetracyclines, etc.) [35], psychiatric medications (tricyclic antidepressants, benzodiazepines, clozapine, and others), heavy metals (copper, lead, arsenicals), antineoplastic drugs (anthracyclines, cyclophosphamide, 5-fluorouracil, tyrosin kinase inhibitors and others), toxic substances (amphetamines, cocaine, opiates), and other toxins (scorpion-bee-wasp stings, snake/spider bites) [36, 37].

### Self-Tolerance and Regulatory Vs. Effector Balance

For many patients with autoimmune myocarditis, no specific direct trigger can be identified. Rather, there appears to be an imbalance in the pro- and antiinflammatory counterparts of the immune system leading to a lack of self-tolerance. Self-tolerance is described as the immune steady-state by which both innate and adaptive parts of the immune system remain unresponsive towards self-antigens [38]. This state is achieved by early T cell progenitor selection and clonal deletion of autoreactive T cell clones [39]. T cell progenitor training takes place in the thymus, where medullary thymic epithelial cells (mTECs) allow for self-antigen expression and stringent central deletion of effector T cells thereafter [40]. In the periphery, mechanisms of regulation and anergy counteract autoreactivity. Studies in humans have shown that cases of autoimmune myocarditis show an increase in peripheral effector T cell levels and an inverse relationship to thymic Treg levels [41]. The development of myocarditis is linked to a shift in peripheral effector T cell and regulatory T cell balance, which is hypothesized to root in a defect in autoimmune regulation within the thymus. Malfunctioning central deletion may lead to escape of autoreactive effector T cells. Furthermore, peripheral effector T cell anergy and peripheral Treg induction are dampened. Failing regulation in both central and peripheral compartments ultimately may lead to a breach in self-tolerance and result in autoreactivity [42].

### Autoreactivity and Genetic Predisposition

As with other autoimmune diseases, studies have tried to elucidate the link between development of autoimmune myocarditis and genetic predisposition. Genetic polymorphism of the major histocompatibility complex (MHC) leads to different binding affinity to antigens, and certain MHC genes are closely associated with the risk of developing certain autoimmune diseases. In humans, HLA (human leukocyte antigen) DR4 has been shown to influence not only the development of myocarditis but also the increased risk of progression to DCM [43]. Besides HLA DR4, also HLA Dr12 and HLA DR15 are positively associated [44]. We have previously shown in a study using transcriptomics in patients with myocarditis vs idiopathic cardiomyopathy that there was higher prevalence of HLA-DQ 1 expression amongst patients with myocarditis vs idiopathic dilated cardiomyopathy [45]. These findings were later supported by Moshlehi and his group, who found higher prevalence of HLA-DQ1 amongst patients with ICI-induced myocarditis [46]. It appears that the HLA-DQ 1 phenotype predisposes to autoimmunity [47], in particular myocarditis.

Genes independent of the MHC such as Eam1 and Eam2 have been associated with myocarditis development in the context of other autoimmune diseases such as lupus and diabetes [48]. With autoreactive T cells being the main driver behind the development of myocarditis, genes that encode for T cell activation and T cell regulation such as CTLA4, PD1, and ICOS can influence the development and severity of autoimmune disease [49].

### Autoreactivity and Antigen Recognition

Apart from genetic susceptibilities, direct malfunctions in antigen presentation and antigen recognition are hypothesized to trigger autoreactivity against myocytes. Molecular mimicry describes the misdirected immunological answer to a self-antigen resembling a non-self-epitope. The recognition by T cells receptors (TCRs) then leads to an immune response against self-antigen. The heavy chain of a myosin isoform (alpha MyHC) has structural similarity with epitopes found on bacteria (bacillus) or fungi (*Cryptococcus neoformans*) [50]. Development of autoimmune myocarditis is discussed to be the outcome of cross reactivity to self-antigen, as seen in patients with cryptococcus infection and resulting myocarditis. Myosin itself is well concealed within the intracellular compartment; its antigen is therefore not expressed on thymic medullary epithelial cells (mTECs) as part of T cell selection. Ischemic or toxic triggers to cardiomyocytes lead to pathologic exposure of intracellular antigens, so called “cryptic antigens” such as myosin heavy chain aMyHC, which in turn launches an autoimmune sensitization [40]. A similar process is thought to start the cascade in the setting of a viral myocarditis whereas T cell clones will lyse virus-laden cardiomyocytes, exposing intrinsic self-antigens to sensitize the immune system. While triggers might be of variable source, the immune sensitization and subsequent T cell infiltration leads to a common pathway of cardiac tissue remodeling and fibrosis, hypertrophy, and apoptosis of cardiomyocytes [51].

### Autoreactivity in Checkpoint Inhibition

Current ICI target one of 3 antigens: cytotoxic T lymphocyte antigen (CTLA4), programmed cell death protein (PD-1), and the ligand of PD-1 (PD-1 L). Inhibition of CTLA4 by IgG antibodies (e.g., ipilimumab) negatively regulates activation of T lymphocytes. PD-1 and PD-1 L inhibitors (e.g., nivolumab, pembrolizumab, atezolizumab, durvalumab) not only activate T cells with “double inhibition” but increase immune tolerance by enhancing apoptosis of antigen presenting cells and reduction of apoptosis of regulatory T cells [2, 52]. Cardiotoxicity as immune-related adverse event has been reported since the establishment of ICI therapy and includes arrhythmias, heart failure, conduction defects, and fulminant myocarditis with fatal outcome [2, 16^•^, 53]. Murine studies demonstrated that cardial PD-1 protects the heart against T cell–mediated inflammation. Subsequently, PD-1 inhibition in mice may induce myocarditis [54]. Furthermore, sequencing of the T cell receptor CD3 shows shared sequences in tumor and cardiac and skeletal muscle suggesting a common antigen response resulting in fatal myocarditis [46]. However, an autopsy study revealed that not all immunologic effects of ICI on the heart become clinically apparent, despite some level of myocarditis with a CD8+ T cell predominant lymphocytic infiltrate on histology [55]. In some reported cases of clinically significant ICI myocarditis, myocardial biopsies showed lymphocytic CD8+ T cells and decreases FoxP3+ regulatory T cells [53].

### MicroRNA in Myocarditis

Microribonucleic acids (miRNAs) are non-coding endogenous small RNA molecules that regulate gene expression on a posttranscriptional level. As such, miRNAs can modulate expression of an entire biological process such as inflammation or fibrosis [56, 57]; therefore, they emerged as epigenetic regulators of myocardial inflammatory response and make them attractive and potential targets for diagnosis and therapy. At least 107 miRNAs have been reported to be involved in viral myocarditis [58–60]. Studies suggest a causal link between miRNA-155, 146b, and − 21 and human coxsackie B3 myocarditis [58]. However, these classic inflammatory miRNAs are not elevated in acute myocarditis [61]. A modulatory effect of miRNA in terms of virulence of cardiotropic viruses has been described. MiRNAs-221/22 regulate virulence and inflammatory pathways in the myocardium [62], and their inhibition leads to aggravated disease. During myocardial injury, miRNA-208 and miRNA-499 are released and thus are evaluated for their prognostic and diagnostic use [61].

Data on miRNA elevation or involvement during ICI-induced myocarditis are currently not available.

## Immunopathogenesis

In physiologic conditions, the cardiac muscle itself harbors only few immune cells [63]. Once an autoreactive sensitization occurs, a diverse set of immune cells and their cytokines migrate into the cardiac interstitial space to launch the inflammatory response [63]. Both cells from the innate and adaptive immune system contribute to a complex interplay of initiation and maintenance of T cell autoreactivity.

### Adaptive Immune System

#### Th1–Th2 Paradigm

While the historic “Th1-Th2” paradigm has expanded with the introduction of other distinct T helper cell subsets, in the setting of autoimmune myocarditis both Th1 and Th2 axes play important roles in maintaining an inflammatory response. Autoimmune disorders as an entity rely on a broad Th1 response mediated by Il–12 to activate CD4+ reactive T cells [63]. Interferon (IFN) gamma, mostly known as mediator to amplify T cell recruitment and proliferation, can increase the severity of cardiac remodeling and fibrosis in the setting of DCM; however, some studies have shown that IFN gamma can influence monocytes and fibroblasts to enhance tissue recovery after inflammatory injury [64]. Contributing to the differentiation of Th1 T cells is tbet, a nuclear factor that has shown more severe myocarditis in tbet knock out rodents [65]. The Th2 response, mediated by Il-4, classically recruits eosinophils from the innate immune system to generate a unique proinflammatory milieu that favors infiltration of eosinophils into the cardiac interstitium, leading to a distinct eosinophilic myocarditis [66]. Proactive cytokine Il4 requires GATA3 and STAT6 as transcription factors to increase the pro inflammatory Th2 axis, while Il13 with its regulatory effects on macrophage differentiation is able to suppress the extent of myocardial inflammation [66].

#### Pro-Inflammatory T Cell Subsets: Th17, Th9, Th22, and Humoral Response

Distinct proinflammatory Th cell subsets are Th17, Th9, and Th22. Especially Th17 has been attributed an important role in the genesis of autoimmune myocarditis as Il–17 has been attributed to facilitate the progression to DCM [67]. While other cells such as NK cells can secrete Il–17, Th17 T cells have been implicated in a variety of chronic inflammatory diseases as well as chronic allograft rejection. Il17 stimulates fibroblast proliferation and has been shown to act in conjuncture with GM CSF secreting fibroblasts to increase severity and progression to cardiac failure [68]. Studies in both rodents and retrospective analysis of human patients have shown that higher levels of Th17 subtype T cells contribute to a progression to DCM [69]. During the acute phase of myocardial inflammation, most of the inflammatory response is concentrated around T cell activation, and patients have no detectable antibodies circulating in the periphery. However, T cell activation leads to T cell and B cell cross talk with subsequent production of specific auto antibodies against cardiac antigens: antibodies against myosin, troponins, and adrenergic receptors that serve as autoreactive epitopes have been classified in patients with ongoing myocardial inflammation. These antibodies are found in high quantities in patient populations progressing to DCM and cardiac failure [70].

#### Anti-Inflammatory T Cell Subsets: Tregs

Regulatory T cells (CD25 + FoxP3+) have been at the forefront of new therapeutic strategies of adoptive cell transfer for a variety of autoimmune diseases [71]. Retrospective studies in patients with myocarditis with progression to DCM have shown that they shared a decreased Treg population while their Th17 population was concomitantly increased [72]. In vitro studies could show that DCM patients with an imbalance of effector and regulatory T cell populations also possess T effector cells that seem less influenced by Treg suppression [73].

Some studies have postulated that the gender disparity seen in viral and autoimmune myocarditis could be associated with the enhanced cross regulation of TLR4 and tim3, leading to enhanced Treg development in females exerting a protective effect and making female patients less prone to induction and severity of myocardial inflammation [74]. These observations could be reproduced in a murine model, in which male mice were found to have more cardiac inflammation and necrosis of cardiomyocytes than their female control group. Apart from their regulatory effect, Tregs are able to specifically influence viral clearing by secretion of TGF beta [72].

### Innate Immune System

The innate immune system does not rely on specific antigen presentation and specific receptors. However, their distinct cell types act on nonspecific toll-like receptors (TLRs). These TLRs, mainly TRL 2, 4, and 5 that are expressed in cardiac muscle, have been shown to initiate NFKB dependent pathway to activate the inflammation cascade to increase monocyte and neutral killer (NK) cells to migrate [75]. We have previously shown that the toll like receptor signaling pathway (TLR 1, 2, and 7) is overexpressed in patients with myocarditis [45]. Fallach and colleagues have shown overexpression of TLR 4 prior to myocardial leukocyte infiltration in mice following septic shock or ischemia [76].

Macrophages (CD68+ CD163+) reside as a pool population in the cardiac tissue and can be further differentiated in three distinct subsets: MHC class II hi /CCR2-, MHC class II low/CCR2- and CCR2+ macrophages [77]. Macrophages of the first two classes contribute to cardiac tissue remodeling and recovery after injury. In the absence of these subsets, cardiac collagen production and tensile strength are decreased leading to fatal rupture. Macrophages linked to increased M2 gene expression can positively induce Treg proliferation which in turn has protective effects by toning down the inflammatory response of the adaptive system [77]. This regulatory pathway could be taken advantage of in a murine model showing cardiac protection with adoptive transfer of M2 polarized macrophages.

Dendritic cells (DCs, CD123+ CD303+) bridge innate and adaptive immune system by acting as antigen-presenting cells (APCs) to T cells. After activation through TLRs, they secrete IFN and induce CD4+ T cell activation and migration into cardiac tissue. Activated dendritic cells also release Il12 and can further increase the proinflammatory milieu by recruiting the Th1 axis [78]. Murine studies underlined this hypothesis by specific induction of autoimmune myocarditis after deliberate dendritic cell transfer loaded with cardiac antigen [78].

Natural killer cells (NK, CD56+ CD94+) have a wide array of protective mechanisms against an overabundance of proinflammation. They inhibit viral laden cardiomyocytes from proliferation, can suppress autoreactive T cells and help monocyte maturation to enhance tissue regeneration. NK cells have been shown to play a major part in recovering from viral myocarditis [79].

## Current Therapy and Future Directions

Treatment of myocarditis includes general nonspecific measures to treat the sequelae of heart disease, including heart failure (HF) therapy and treatment of arrhythmias according to current guidelines and scientific statements [3]. Mechanical circulatory support and transplantation may remain a potential last resort for patients with refractory heart failure despite optimum medical therapy.

Furthermore, therapy for viral myocarditis has been focusing on specific antiviral treatment, while non-viral autoimmune myocarditis has been treated with broad band immunosuppressive agents and some immune modulating drugs [3]. Immunosuppression appears mandatory in specific noninfectious settings of acute myocarditis such as giant cell myocarditis, necrotizing eosinophilic myocarditis, and cardiac sarcoidosis.

However, the shared end pathway of inflammation, tissue remodeling, fibrosis and progression to DCM, and cardiac failure make both immune suppression and immune modulation valid therapeutic options for a wide array of myocarditis subtypes.

Immunosuppression targets general inflammation and is achieved classically with corticosteroids, cyclosporine A, azathioprine, or a combination of the aforementioned. Most data of immunosuppressive therapy have been obtained using corticosteroids alone, or in combination with azathioprine or cyclosporine A. Several randomized controlled trials provide results for prednisone and azathioprine at 3 months [80], 6 months [81], and 1 year [82], for prednisone alone at 3 months [83], and for combined prednisone, cyclosporine A, or azathioprine at 6 months [84]. However, differences in patient selection and study design, as well as focus on LVEF as primary endpoint on relative short follow-up durations, have led to contradicting results. A recent retrospective analysis by Merken and colleagues revealed a potential beneficial effect of immunosuppression in patients with myocarditis [85]. A prospective multicenter trial using azathioprine and prednisone is currently ongoing (NCT01877746). In giant cell myocarditis, immunosuppression including corticosteroids, cyclosporine, and the possible addition of azathioprine is the main treatment strategy. Sudden interruption of immunosuppression in these cases within the first 2 years has been associated with fatal relapse of the disease [86].

In the event of immunotherapy-induced myocarditis (e.g., by checkpoint inhibitors), the assumption of an immune antigen reaction similar to an allograft rejection or rheumatic disease, vindicates the use of immunosuppression with corticosteroids. However, until now only case reports and case series have been published to guide this conclusion [87]. Additional immunosuppressive therapy with mycophenolate mofetil or calcineurin inhibitors may be beneficial. Successful treatment with equine antithymocyte globulin in ICI-related and corticosteroid-resistant myocarditis has been reported [88].

To further affect myocarditis via immune modulation, uncontrolled studies on intravenous immunoglobulin therapy showed promising results for improved recovery of LVEF [89, 90]. However, a randomized investigation (IMAC trial) showed no effective outcome, but only 15% of patients had biopsy-proven myocarditis of non-specified cause in this study [91]. Immunoadsorption and plasmapheresis aims to lower cardiotoxic antibodies and immune complexes in the plasma, and reported effects of small randomized studies are promising [92, 93].

Immune modulating agents allow for a more targeted approach with reduced side effects. An anti-CD3 monoclonal antibody (muronumab) suppresses lymphocyte activation and proliferation. IL-6 antibody blocks the Il-6 receptor and has been shown to be beneficial in viral myocarditis [94]. The involvement of T cell to B cell crosstalk and the emergence of specific anti-cardiac antibodies related to severe cases of myocarditis give room for more targeted therapies than the simple elimination of antibodies via plasmapheresis or immunoadsorption. Costimulatory blockade, the inhibition of CD28 and B7 T cell receptors has been shown to reduce T cell proliferation, B cell activation, and sensitization via circulating antibodies [95]. The concept of costimulatory blockade has been applied to tolerance induction models in non-human primates and has shown prolonged graft survival with decreased CD4+ T cell activity [95]. Similar approaches target the CD28 receptor itself with belatacept, a humanized antibody that has proven to be beneficial in renal transplant recipients [95].

These single agents target specific cells and/or a specific cytokine milieu. A broader, more conceptual approach has been adopted from the field of transplantation: the induction of tolerance aims to restore a stable balance between effector and regulatory forces, suppress autoreactivity, keep overabundant T cell activity in check and enhance myocardial tissue repair [38, 96].

Studies of the T cell repertoire were able to show an association of specific clonal deletion of T cells in tolerant patients after bone marrow transplantation [97]. Transgenic murine models of myocarditis that had myosin laden antigen presented on mTECS in the thymus were able to clonally delete autoreactive T cells and were protected from myocarditis induction [40]. Further studies and the following of T cell clones are needed in order to harness clonal deletion for myocarditis therapy.

Regulatory failure leads to imbalance of effector and regulatory T cells. The idea of restoring the said balance has been attempted by increasing the peripheral Treg pool. Studies on tolerance induction in solid organ transplants in non-human primates have harnessed the adoptive transfer of ex vivo expanded, autologous Treg infusions to alter T effector vs T regulatory balance in favor of a less inflammatory setting [98, 99]. Ongoing trials on tolerance induction in kidney graft recipients with the help of Treg infusions in humans underline the feasibility and potential of ex vivo expanded, recipient targeted adoptive cell transfer [100–102]. Understanding the underlying mechanism of innate and adaptive immune players at different stages of autoimmune myocarditis is crucial to further develop targeted therapies.

## Conclusion

Myocarditis continues to be an underdiagnosed inflammatory disease of the heart of which its pathophysiology has not been fully understood as of today. Limited availability and accuracy of current diagnostic standards for myocarditis continue to be a major public health concern, as myocarditis is frequently found during autopsy in young adults who died of sudden cardiac death, signaling that our diagnostic screening has to improve. Improved diagnostic accuracy is the foundation for a better understanding of the disease, its clinical trajectory and for better therapies consequently. Many experts currently suggest that the most common etiology of myocarditis in the Western World is an autoimmune reaction initially triggered by an inciting event, such as a viral infection. As for other autoimmune diseases, the etiology may also be spontaneous imbalance of T cell subsets leading to inflammation. Lately, immunomodulatory drugs such as ICI have been shown to induce myocarditis in certain patients through disinhibition of the immune system. Genetic predisposition appears to play a role. Interpretation of data from clinical trials investigating therapies in myocarditis has been limited overall due to low numbers of participants. International collaborations will help to collect sufficient data to provide clear guidance for physicians in the future on how subtypes of myocarditis should be treated and monitored.
